# Supplementary material for: Holotype genome of the lesula provides insights into demography and evolution of a threatened primate lineage
Source: Genome Biol. 2025 Dec 1;26:408. doi: 10.1186/s13059-025-03877-z (PMC12667059; doi:10.1186/s13059-025-03877-z)
Supplement: Supplementary file 2 — Additional file 2. Supplementary figures (Fig. S1-S9). [file 13059_2025_3877_MOESM2_ESM.docx]

# Holotype genome of the lesula provides insights into demography and evolution of a threatened primate lineage

Jensen et al.

Supplementary figures Fig. S1-S9


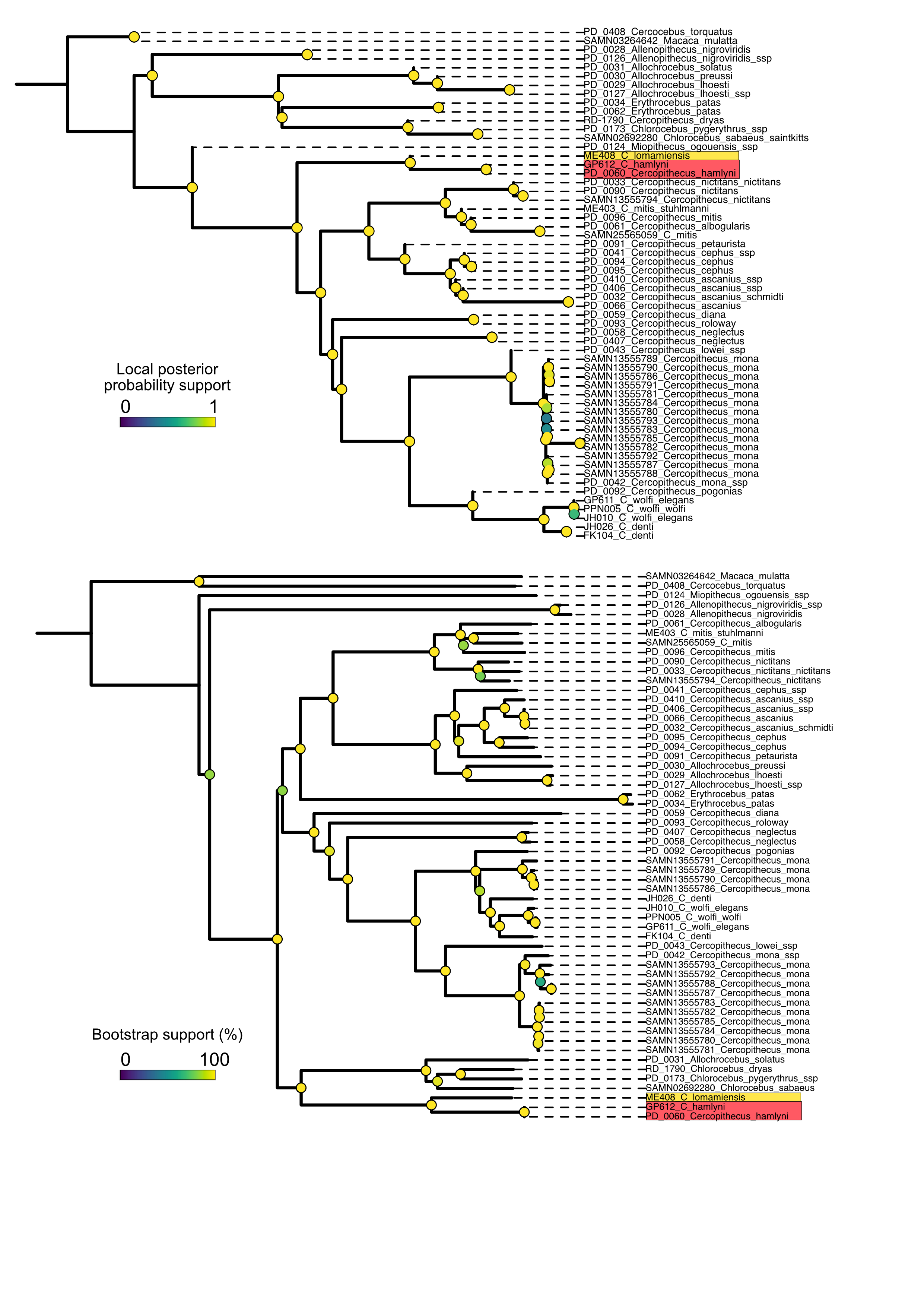


**Fig. S1**. Autosomal coalescent-based tree estimated with ASTRAL (top) and mito­chondrial maximum likelihood tree estimated with IQTree (bottom) with all samples included.

**Fig. S2.** Comparison of divergence time estimates based on different data types and fossil calibration points. **A)** Cladogram of the phylogeny for which divergence times was estimated. Filled circles with numbers highlight nodes for which fossil calibration times were available from de Vries and Beck (2023; these fossil calibrations were used in our main analysis, Figure 1B), and the open circle the fossil calibration from Hart et al. (2012; when this fossil calibration was used, we also applied a soft constraint on the root of the tree at <15 Mya [see Methods]). The fossil calibration intervals are detailed in the boxes to the right. Node numbers are shown on internal nodes (numbers without circles). **B)** Divergence time estimates from four different combinations of sequence data and fossil calibration points. Violin plots show the distribution of the mcmc samples, with the mean age highlighted by points. Node numbers correspond to those in (A), and the split between *C. lomamiensis* and *C. hamlyni* (node 43) is highlighted, with the numbers above the plots showing the mean age estimates of the different runs. The 10*10 loci analyses were run on 10 sets of 10 autosomal loci á 5 kb, as in our main analysis (Figure 1B in the main text), whereas the Xq13.3 locus is a 4,621 bp long X-linked, intergenic locus that was also used in Hart et al. (2012).


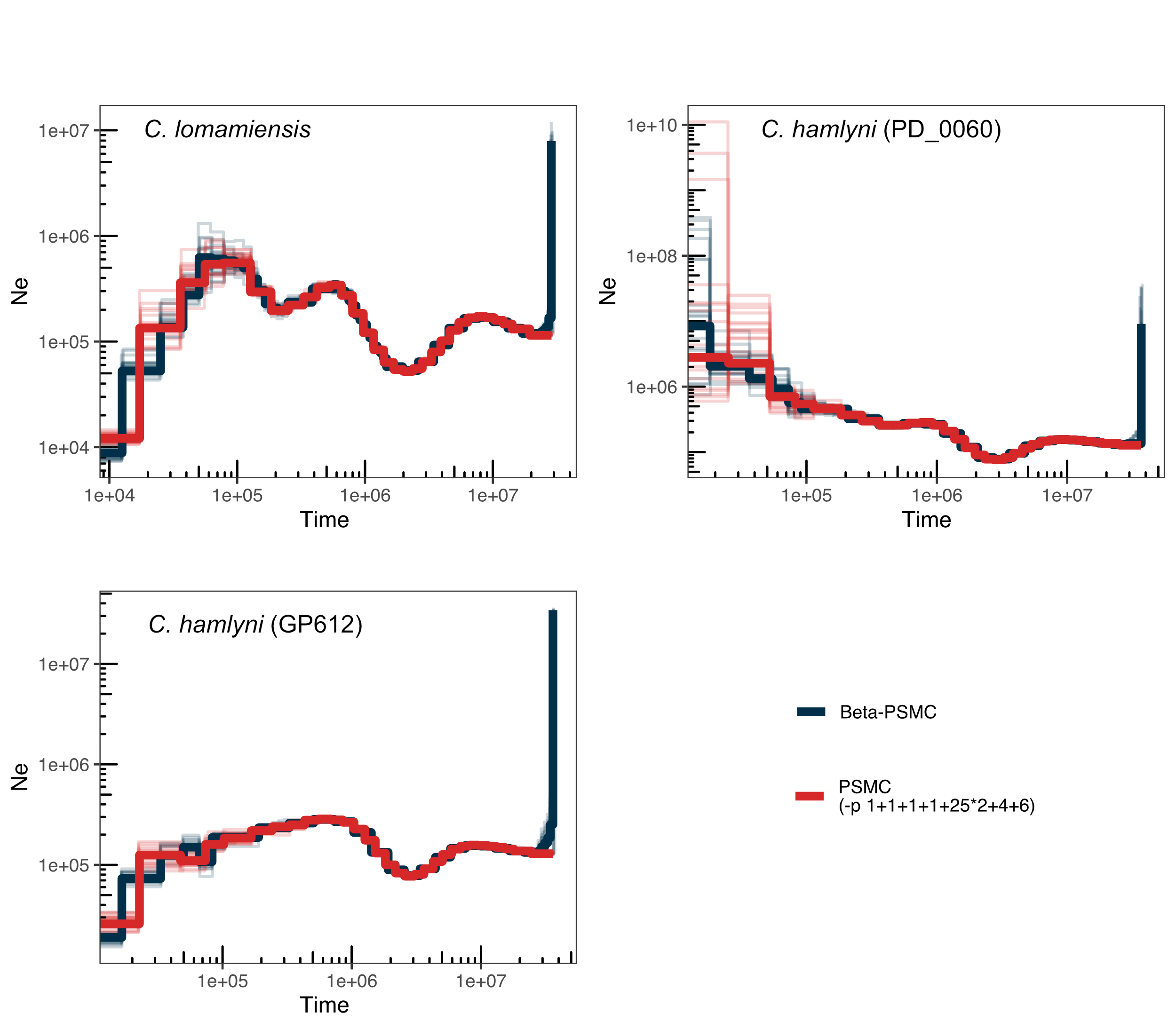


**Fig. S3.** Effective population size through time estimated with beta-PSMC and PSMC, with the latter using parameter settings shown to decrease biases in recent times (Hilgers et al., 2024).

**Fig. S4.** Correlation between total number of runs of homozygosity (ROH) and their summed length in all *Cercopithecus* and *Miopithecus* samples.

**Fig. S5.** Heterozygosity of SNPs with different predicted impacts in *C. lomamiensis* and *C. hamlyni*.

**Fig. S6.** Alignments of codon 197 of the gene *POMC* (nucleotide sequence to the left, translated amino acid sequence to the right), inferred to evolve under positive selection in the *C. hamlyni* lineage. *C. hamlyni* experienced two nucleotide substitutions in this codon (G->A in second position and C->T in third position), leading to an amino acid substitution (A [alanine] -> M [methionine]). Both *C. hamlyni* individuals carry the identified substitutions.

**
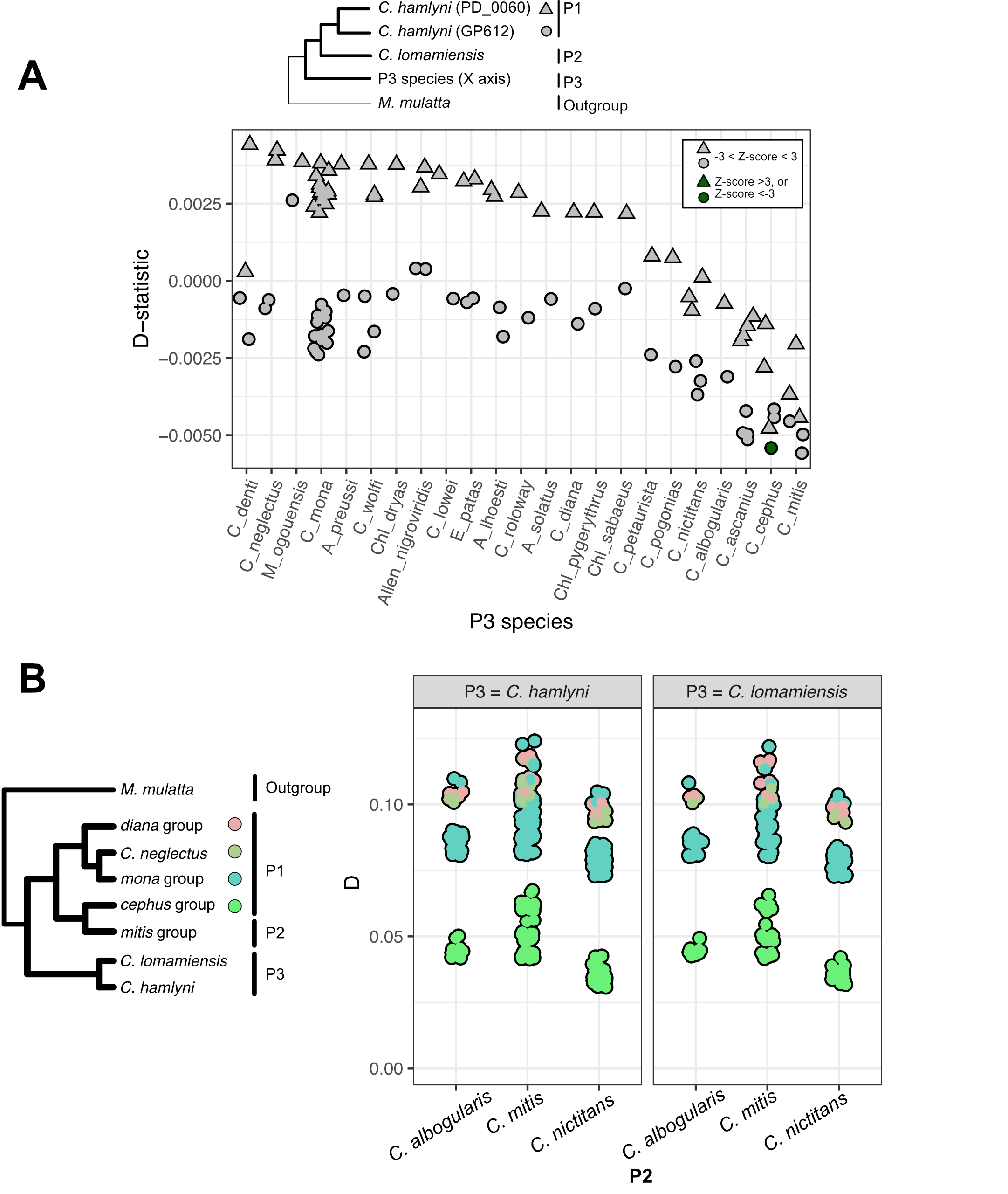
Fig. S7.** A) Excess allele sharing (D-statistics) between *C. lomamiensis* and all other guenon samples, relative to the two *C. hamlyni* samples (circles and triangles). Green circles/triangles indicate estimates that deviate significantly from 0 (Z-score > 3/Z-score < -3). B) Excess allele sharing (D-statistics) between *C. hamlyni/C. lomamiensis* and *mitis* group lineages. Highly similar estimates confirm that the gene flow between *C. mitis* and the *hamlyni* group occurred prior to the split between *C. hamlyni* and *C. lomamiensis*.


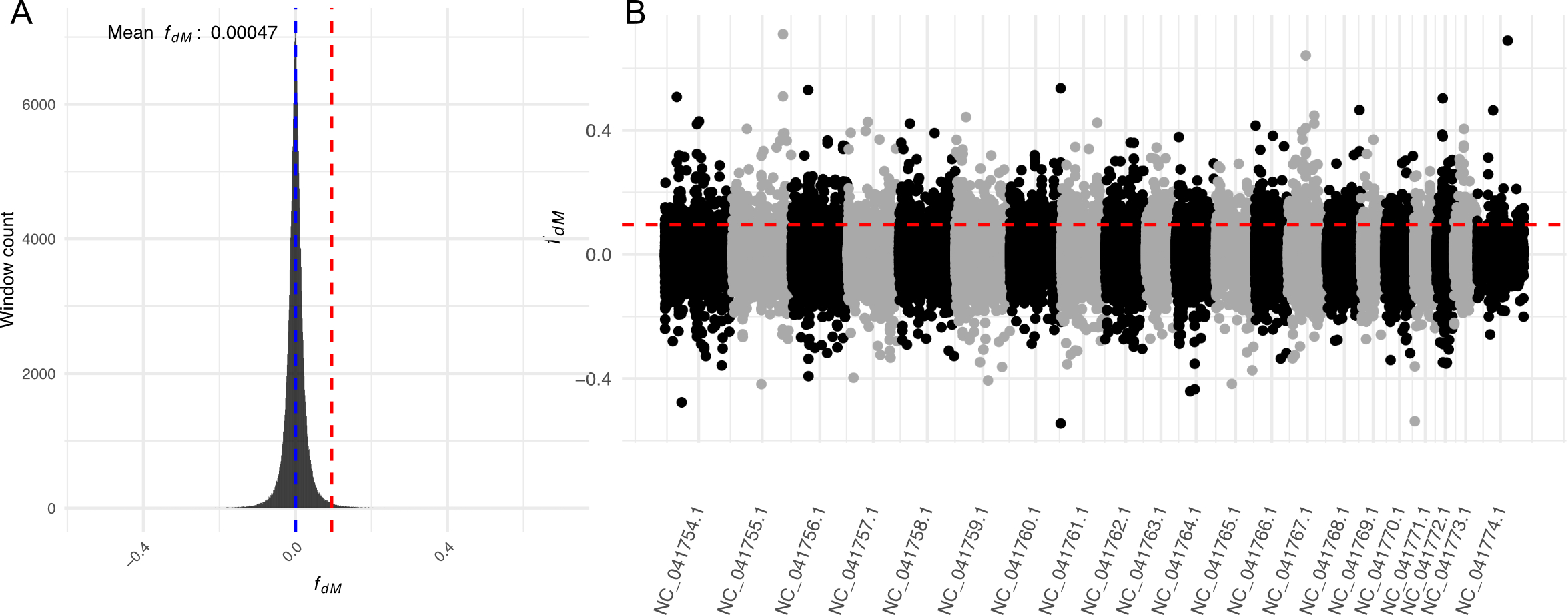


**Fig. S8.** Genomic landscape of shared excess allele sharing between the *hamlyni* lineage and the terrestrial clade, relative to other *Cercopithecus* species (P1: *Cercopithecus* spp., P2: *hamlyni* group spp., P3: terrestrial clade spp., Outgroup: *M. mulatta*). A) Shows the distribution of *f_dM_* estimates in 10 kb windows, with the blue dashed line highlighting the genome-wide mean, and the red dashed line the 99^th^ percentile. B) shows *f_dM_* estimates along the reference genome, with the dashed line highlighting the 99^th^ percentile.


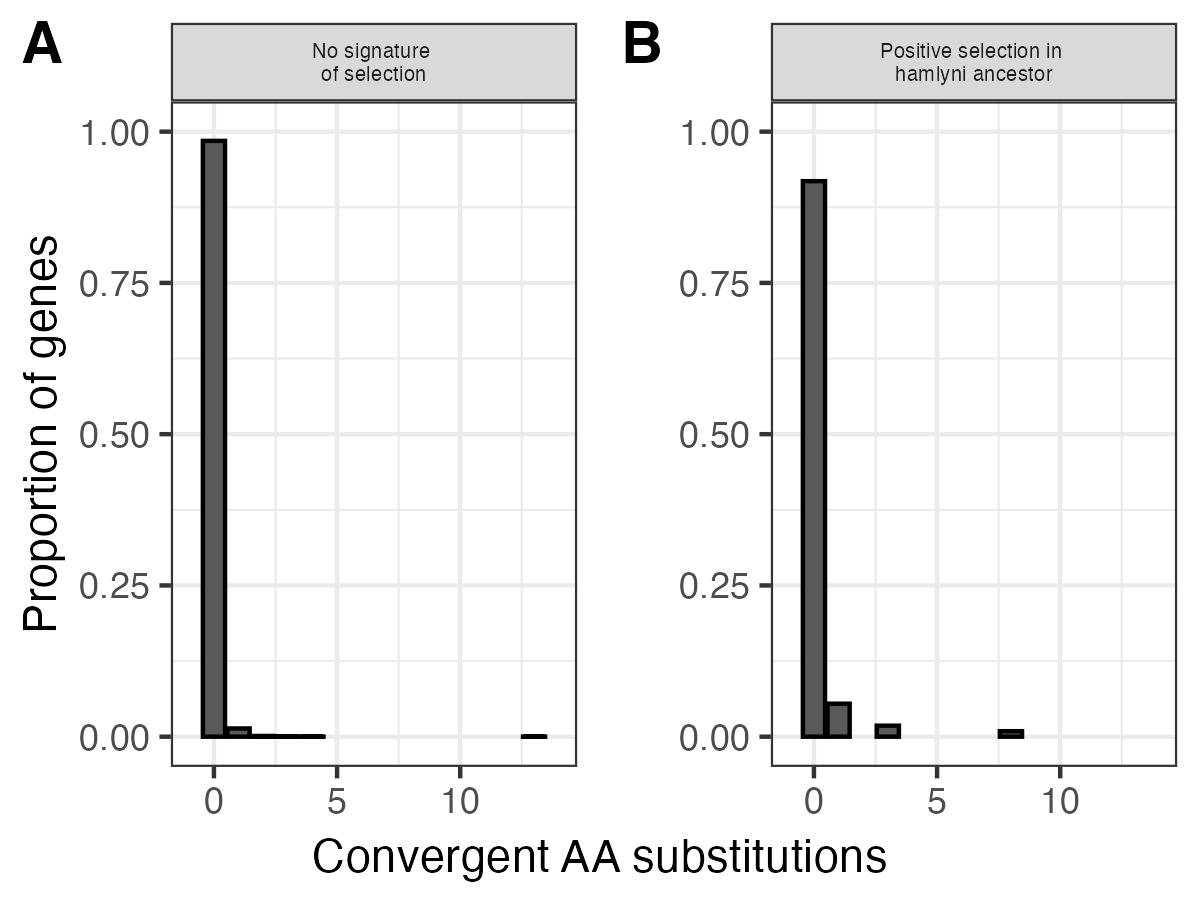


**Fig. S9**. Proportion of genes with *X* number of convergent AA substitutions be­tween the terrestrial clade linage and the *hamlyni* group, in (A) genes without any signature of selection and (B) genes with signatures of positive selection in the *hamlyni* ancestor, based on HyPhy analyses.
